# Skeletal stability of inter-molar mandibular distraction osteogenesis in growing patients

**DOI:** 10.1186/s40510-023-00507-x

**Published:** 2024-02-26

**Authors:** Matthew Lewis, Peter Lewis, Tung Nguyen, Alethea Rea, Mithran S. Goonewardene

**Affiliations:** 1 Orthodontics, Oral Health Centre of Western Australia, School of Dentistry, The University of Western Australia, 17 Monash Avenue, Nedlands, WA 6009 Australia; 2 Private Practice in Orthodontics, 5 Khartoum Avenue, Gordon, NSW 2072 Australia; 3 Department of Orthodontics, University of North Carolina, CB# 7450, 201 Brauer Hall, Chapel Hill, NC 27599-7450 USA; 4 UWA Centre for Applied Statistics, School of Mathematics and Statistics, The University of Western Australia, 35 Stirling Highway, Nedlands, WA 6009 Australia; 5 Orthodontics, Oral Health Centre of Western Australia, School of Dentistry, The University of Western Australia, 17 Monash Avenue, Nedlands, WA 6009 Australia

## Abstract

**Introduction:**

The aim of this retrospective study was to firstly assess the stability of surgical advancement using inter-molar mandibular distraction osteogenesis (IMDO) and secondly to assess the impact of the surgical intervention on subsequent mandibular growth in patients with residual growth.

**Methods:**

The sample consisted of 17 (13F and 4M) consecutively treated patients who underwent IMDO and orthodontic treatment. Cephalometric analysis was performed at three time points: T0 prior to distraction; T1 post-distraction immediately prior to surgical removal of the distractors; and T2 following completion of orthodontic treatment when the final lateral cephalogram was taken (0.86–4.37 years after T1). Statistical comparison of lower facial height, mandibular length, growth, condylar position and anterior mandibular rotation was performed.

**Results:**

No association was found between changes in any of the cephalometric measurements and the length of the follow-up interval. The anterior mandibular segment underwent clockwise rotation during distraction and recovered to near its pre-distraction angulation during remodelling. An increase in the lower facial height of 1.88 ± 2.81mm also occurred during distraction (T0–T1) and was maintained during the follow-up period (T1–T2). Post-distraction (T1–T2) growth of lower facial height (*p* value 0.872) and mandibular length (*p* value 0.251) showed no association when compared to an untreated control group and an overall reduction in growth was reported.

**Conclusions:**

IMDO was highly stable within a follow-up period of 2.3 ± 0.9 years; however, growth appears to have been inhibited.

## Introduction

Surgical correction of severe skeletal disharmony is often required to achieve a desirable facial and occlusal outcome [1, 2]. A deficient mandible has been shown to be a predominant feature of the Class II malocclusion, [3] which may be addressed by surgical advancement. The bilateral sagittal split osteotomy (BSSO) is the most common surgical technique for surgical correction of mandibular deformities [4]. Although the BSSO has gained general acceptance, several limitations have been associated with this procedure including: surgical relapse; length of achievable correction; risk of degenerative condylar changes; acute soft tissue stretch; and interference in residual growth in growing patients [5–12].

Mandibular distraction osteogenesis is a biological process by which the surfaces of an osteotomised bone. This concept was first introduced by Codivilla and the technique refined based on sound mechanical and biological principles by Ilizarov [13–15]. A osteoid-like callus forms that is gradually separated to generate new bone. This has been proposed as a less invasive surgical technique and has been reported to achieve significant aesthetic and functional improvement. Rosenthal first reported application of a tooth borne appliance to progressively elongate an osteotomised mandible in 1927 [16]. However, the popularity of the BSSO reduced the routine application of distraction osteogenesis from 1955 [4]. Since the first contemporary publication by Michieli and Miotti in 1977 [16], McCarthy pioneered the progression of distraction osteogenesis in the craniofacial skeleton, with numerous applications in all three dimensions [17]. Moreover, design modifications have resulted in development from an extra-oral fixation mechanism, with associated soft tissue shortcomings to less-invasive complex intra-oral devices. Distraction osteogenesis, has been proposed as a surgical technique which may reduce many of the additional shortcomings of conventional osteotomies and has been recommended for earlier surgical correction for some severe dentofacial deformities such as Pierre Robin sequence and hemifacial microsomia [10, 11].

Distraction osteogenesis, for the correction of Class II malocclusion has been proposed to be performed in either the ramus or body of the mandible [18]; however, vertical lengthening of the mandibular ramus has been shown to be highly unstable [19]. Distraction osteogenesis in the body of the mandible through bilateral vertical osteotomies are located posterior to the final molar, which mimics the movements of a BSSO and has been reported to have minimal adverse effects on the mandible including maintenance of the occlusal plane [17].

Distraction osteogenesis has presented several challenges to clinicians including risk of infection, pain during activation, damage to the soft tissues and failure of fixation resulting in aberrant vectors of movement. Moreover, healing issues may present including non-union or early consolidation [20–22]. Clinicians therefore may select distraction osteogenesis for corrections of greater magnitude (> 10 mm) because of the limitations of soft tissue compliance and stability associated with the acute movement encountered in routine orthographic surgery. The gradual lengthening of the mandible that encourages histiogenesis of the various tissues such as muscles, tendons, fascial sheaths, blood vessels and nerves decreases the factors associated with relapse [23, 24].

Inter-molar mandibular distraction osteogenesis (IMDO) is a novel technique for mandibular distraction in which the bilateral vertical osteotomies and distraction sites are located between the first and second mandibular molars [25, 26]. In a traditional BSSO case, pre-surgical orthodontics often requires the removal of dental units to allow for surgical decompensation. The advantage of IMDO is that the location of the surgical site may be consistently reproduced to generate bone formation and spacing between the molars to be utilised for traditional orthodontic mechanotherapy such as alignment of crowded arches and/or to remove dental compensations from the pre-surgical morphology. In this study, IMDO was performed for the correction of severe Class II skeletal discrepancies in growing individuals.

The aim of this retrospective study was to firstly assess the stability of the surgical advancement using IMDO and secondly to assess the impact of the surgical intervention on subsequent mandibular growth as the patients had been primarily treated prior to growth cessation.

## Method

Ethics approval was attained from the Human Research Ethics Committee at the University of Western Australia. Signed informed consent was received obtained from all patients whose records were used in this study.

### Sample population

This retrospective study assessed circum-adolescent consecutive patients who underwent IMDO treated by a single orthodontist (PL) and a single oral maxillo-facial surgeon (PC). Consent to use the patients’ records for research purposes was obtained prior to the commencement of the distraction surgery. The records of these consecutive patients who underwent T0 between March 2013 and September 2015 were assessed for eligibility.

**Fig. 1:**
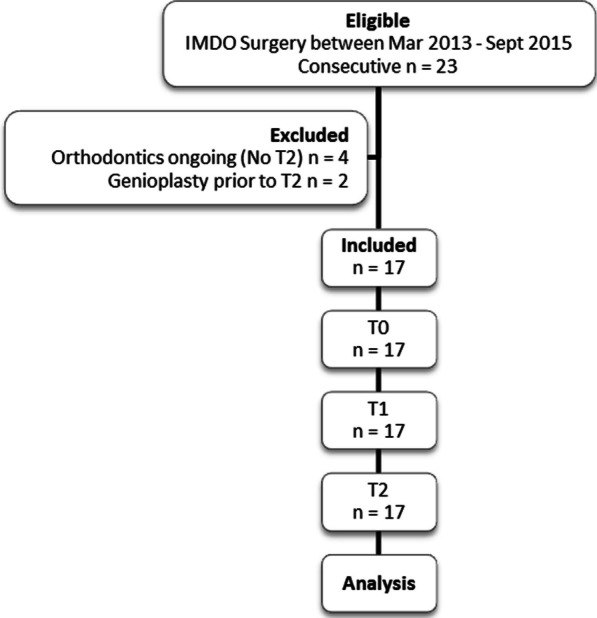
Flowchart describing the protocol for patient inclusion within this study

The patients’ treatment records included radiographic images at three time points: T0 prior to distraction; T1 post-distraction immediately prior to surgical removal of the distractors; and T2 following completion of orthodontic treatment. At T0 and T1, low-dose spiral CTs recorded in a specialist radiologist’s office were completed as part of the routine treatment of the surgery to assess the surgical site and to ensure skeletal fusion, respectively. The low-dose spiral CT radiographs from T0 and T1 were imported into SimPlant O&O (V3.0.0.59) by Materialise Dental to produce a constructed lateral cephalogram. At T2 a post-orthodontic lateral cephalogram was taken in one of the orthodontist’s two offices to assess the confluence of the bony healing. All the radiographs collected were produced as a component of the routine surgical and orthodontic treatment of these patients. The records of 17 (13F and 4M) patients were found to be complete and were included within this study. Six patients were excluded as shown in Fig. 1.

**Fig. 2:**
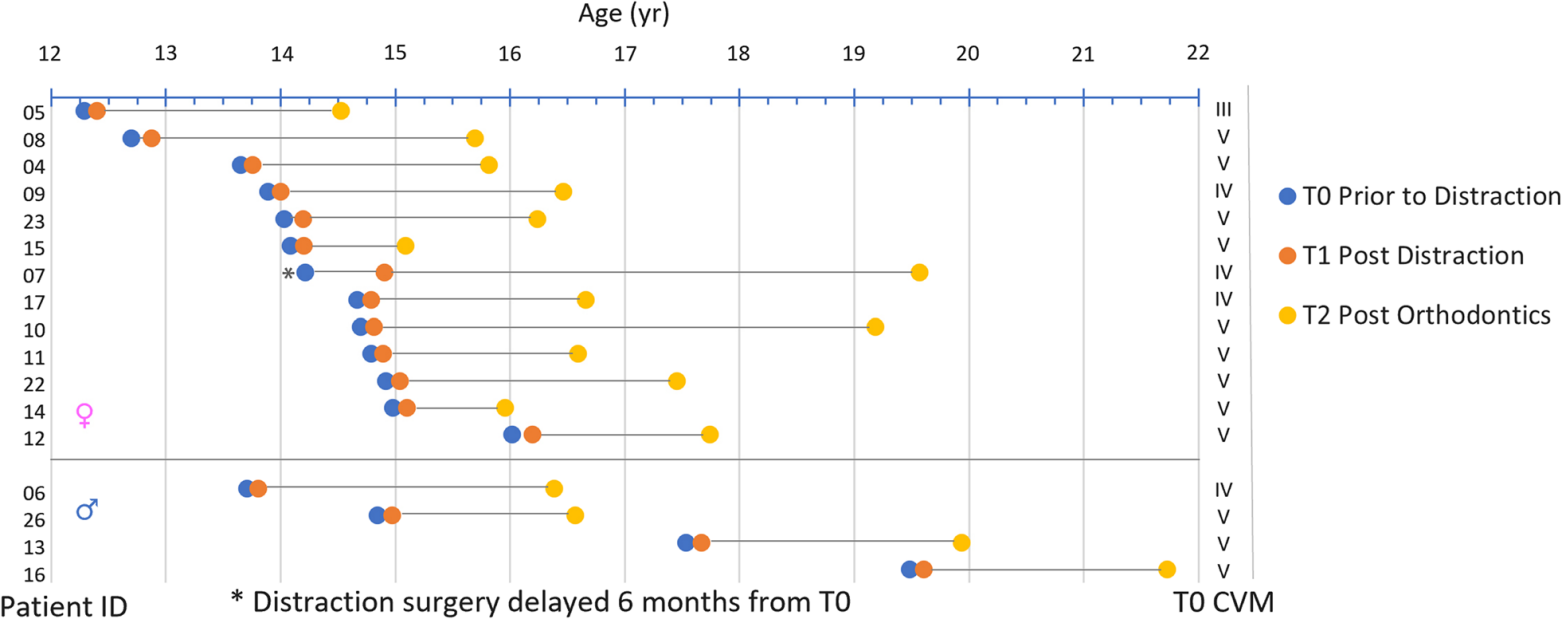
Radiographic and surgical dates relative to age and gender for each patient

The selection criteria for commencing IMDO included an Index of Orthognathic Functional Treatment Need index (IOFTN) of 4 or greater [27], with severe mandibular retrognathia with an ANB angle greater than 7°; an overjet greater than 8mm; or a molar relationship greater than a full unit Class II. The observation intervals, the cervical vertebrate maturation stages and the cephalometric data are summarised in Tables 1 and 2. Figure 2 represents a summary of the radiographic and surgical dates relative to age and gender of each patient.

**Table 1:** Observation intervals, gender and CVM distribution

Observation intervals	Mean	SD	Gender		CVM	
Age T0 (yr)	14.73	1.7	Female	13	III	1
			Male	4	IV	4
Distraction (Surgery-T1) (yr)	0.13	0.02			V	12
Orthodontics (T1–T2) (yr)	2.26	1

**Table 2:** Mean changes in cephalometric angles, linear measurements, horizontal changes (x-axis) and vertical changes (y-axis) between T0–T1, T1–T2 and T0–T2

	Distraction osteogenesis (T0–T1)	Orthodontics (T1–T2)	Treatment (T0–T2)
	Mean (SD)	Mean (SD)	Mean (SD)
*Angular changes*			
SNA	0.06 (0.27)	0.11 (1.05)	0.17 (1.05)
SNB	4.74 (1.10)	− 0.39 (1.22)	4.35 (1.53)
ANB	− 4.46 (1.03)	0.47 (1.15)	− 4.19 (1.36)
MxMd	0.47 (2.29)	1.03 (2.46)	1.50 (3.18)
MPA	0.46 (2.07)	− 0.34 (2.05)	0.12 (2.78)
AMA	7.47 (2.96)	− 5.94 (2.66)	1.53 (1.82)
*Linear changes*			
Mx (CoA)	1.14 (1.41)	− 0.44 (2.18)	0.69 (1.66)
Md (CoGn)	7.08 (1.58)	− 0.41 (3.50)	6.66 (2.77)
LFH (ANS-Me)	1.88 (2.81)	0.34 (2.46)	2.22 (3.30)
*Horizontal changes (x-axis)*			
S	− 0.04 (0.18)	− 0.02 (0.44)	− 0.06 (0.47)
ANS	0.03 (0.25)	0.70 (1.39)	0.73 (1.44)
PNS	0.02 (0.41)	− 0.36 (2.47)	− 0.34 (2.38)
A	0.02 (0.12)	0.16 (0.81)	0.18 (0.84)
Co	− 1.21 (1.52)	0.88 (2.25)	− 0.33 (1.66)
Go	− 0.81 (2.14)	− 0.46 (1.64)	− 1.27 (1.63)
B	7.33 (1.75)	− 0.55 (1.82)	6.78 (2.47)
Pg	5.84 (2.09)	− 0.12 (2.05)	5.71 (2.84)
Gn	5.34 (2.15)	0.00 (2.24)	5.34 (2.66)
Me	5.21 (2.45)	0.01 (2.30)	5.21 (2.84)
*Vertical changes (y-axis)*			
S	0.06 (0.26)	− 0.08 (0.52)	− 0.02 (0.52)
ANS	− 0.12 (0.23)	− 0.05 (1.01)	− 0.17 (0.95)
PNS	− 0.05 (0.48)	− 1.32 (1.45)	− 1.36 (1.36)
A	− 0.17 (0.35)	− 0.60 (1.04)	− 0.77 (1.06)
Co	− 0.35 (1.15)	0.41 (2.37)	0.06 (2.34)
Go	− 0.22 (2.09)	− 0.56 (2.69)	− 0.78 (2.84)
B	− 4.37 (3.14)	0.72 (3.71)	− 3.65 (4.28)
Pg	− 3.23 (2.63)	0.40 (3.13)	− 2.83 (2.84)
Gn	− 3.90 (2.35)	0.21 (2.70)	− 3.69 (3.11)
Me	− 3.63 (2.47)	− 0.18 (2.73)	− 3.81 (3.28)

A power analysis of sample size calculation is based on the primary outcome that surgery impacts growth of the mandible (CoGn). Class II control growth of patients aged 14.3–15.4 years has been measured to be 1.9 mm with a standard deviation of 1.1 mm [28]. Therefore, to measure a 2-mm impact of surgery, we required a sample of at least 6 to be able to reject the null hypothesis that surgery makes no difference with a power of 0.8.

### Pre-surgical orthodontic protocol

**Fig. 3:**
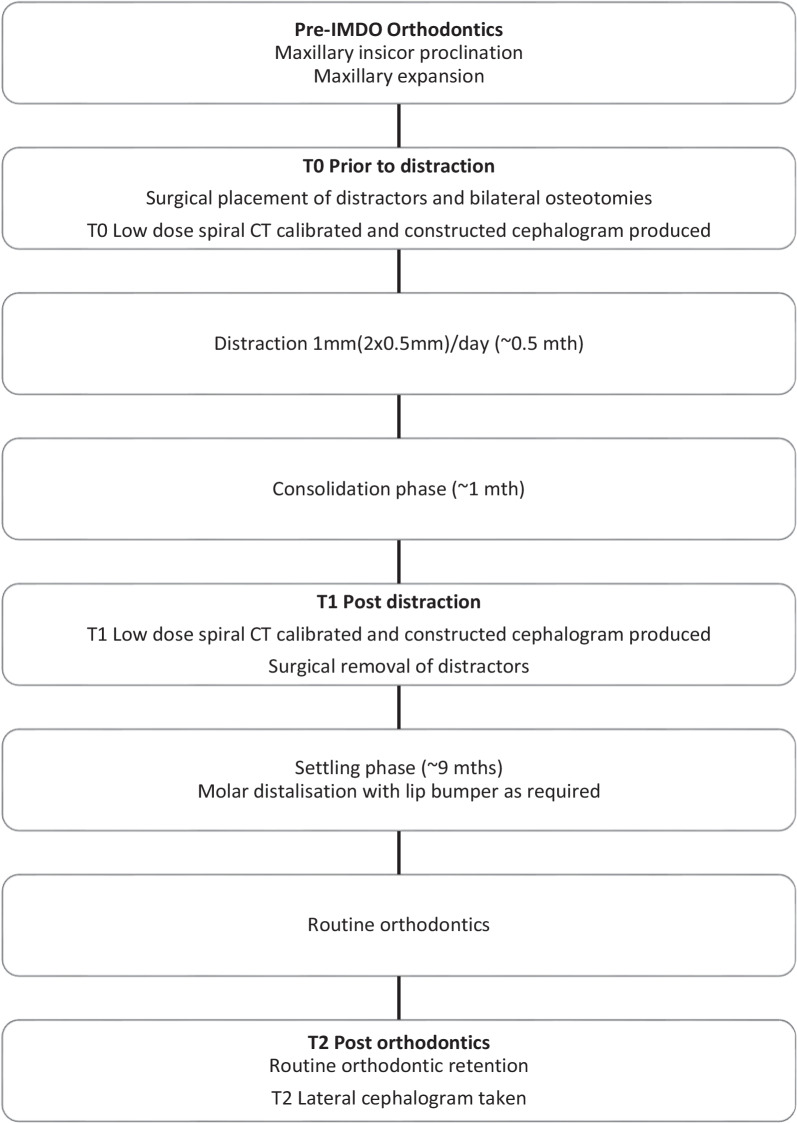
Flowchart describing the IMDO orthodontic and surgical treatment protocol

All patients within this study were treated following a standardised orthodontic and surgical protocol (Fig. 3). The aim of the pre-surgical orthodontic treatment was the correction of any transverse discrepancies and proclination and protrusion of the maxillary incisors in order to remove any dentoalveolar interferences with the subsequent mandibular advancement. When required, additional spacing was created distal to the maxillary anterior teeth to allow an even greater anteroposterior mandibular correction. Following the completion of these pre-surgical anteroposterior corrections, IMDO was performed. The transverse maxillary correction was either performed prior to, or during, the IMDO therapy.

### Surgical protocol

**Fig. 4:**
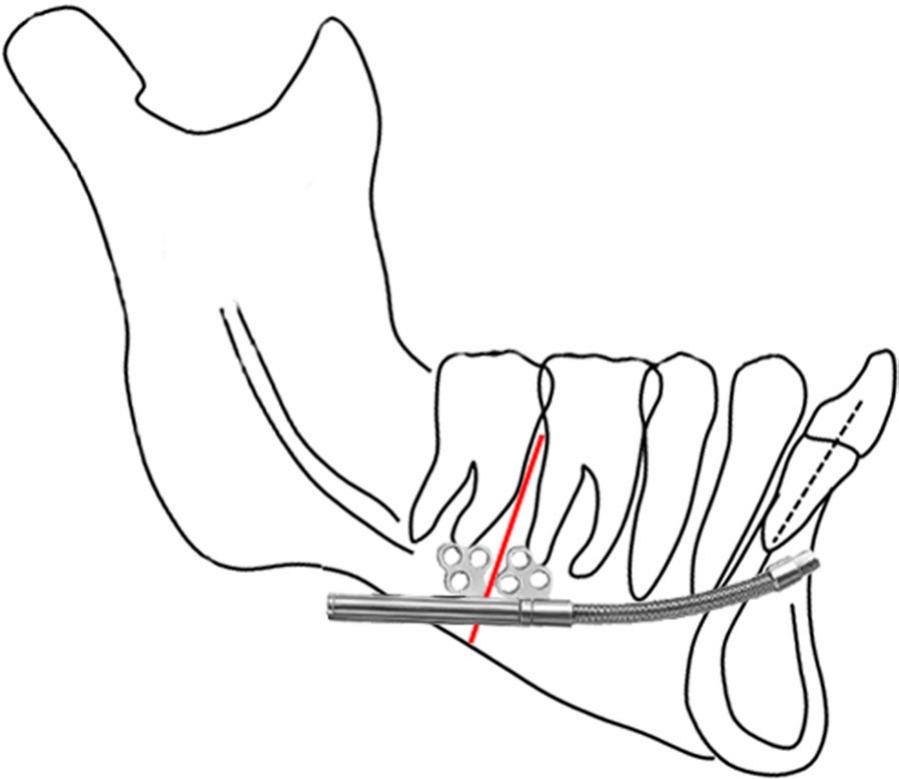
Diagrammatic representation of the osteotomy site (red) and modified KLS Martin distractor

The surgical protocol for the IMDO commenced by raising a buccal muco-periosteal flap along the gingival margin from the distal third of the canine to the retromolar pad. In IMDO, the bilateral vertical osteotomies and the distraction sites were located between the first and second mandibular molars (Fig. 4). A tapered fissure burr was then utilised to place the osteotomy to the depth of the cortex, and a lingual notch was placed on the inferior border of the mandible. Then, a sharp Epker osteotome was utilised to notch the uncut crestal bone closest to the molar teeth.

Once the outer osteotomy line had been finalised, the distractors were aligned over the osteotomy line at the classic 30 degree rise and at the height of the external oblique ridge. The distractor was predrilled to facilitate fixation after completing the osteotomy. Two modified KLS Martin Zurich distractors were utilised, and the distractor arms were placed to exit through the anterior oral mucosa to facilitate activation. This allowed the entire appliance to be placed intra-orally without extra-oral facial scarring [18].

Application of the Smith’s Spreader allowed for controlled crack propagation to occur between the molars, and then, an IMDO Spreader was utilised to complete the circum-cortical mobilisation, particularly in the infero-medial cortical region. The distractors were then reapplied utilising the predrilled holes and screws placed. 7-mm screws were utilised except for the anterior superior screw which was 5mm to prevent damage to the first molar distal root. Finally, the distractors were tested with 8–10 full test turns to ensure mobilisation prior to sutural closure.

Distraction was performed at a rate of 1/2 mm twice daily per side until the desired advancement for each side was achieved based upon clinical examination. Differential amounts of advancement were performed for correction of any anteroposterior mandibular skeletal asymmetry. The distractors were removed on average 1.5 months following the distraction surgery.

### Post-distraction orthodontics

**Fig. 5:**
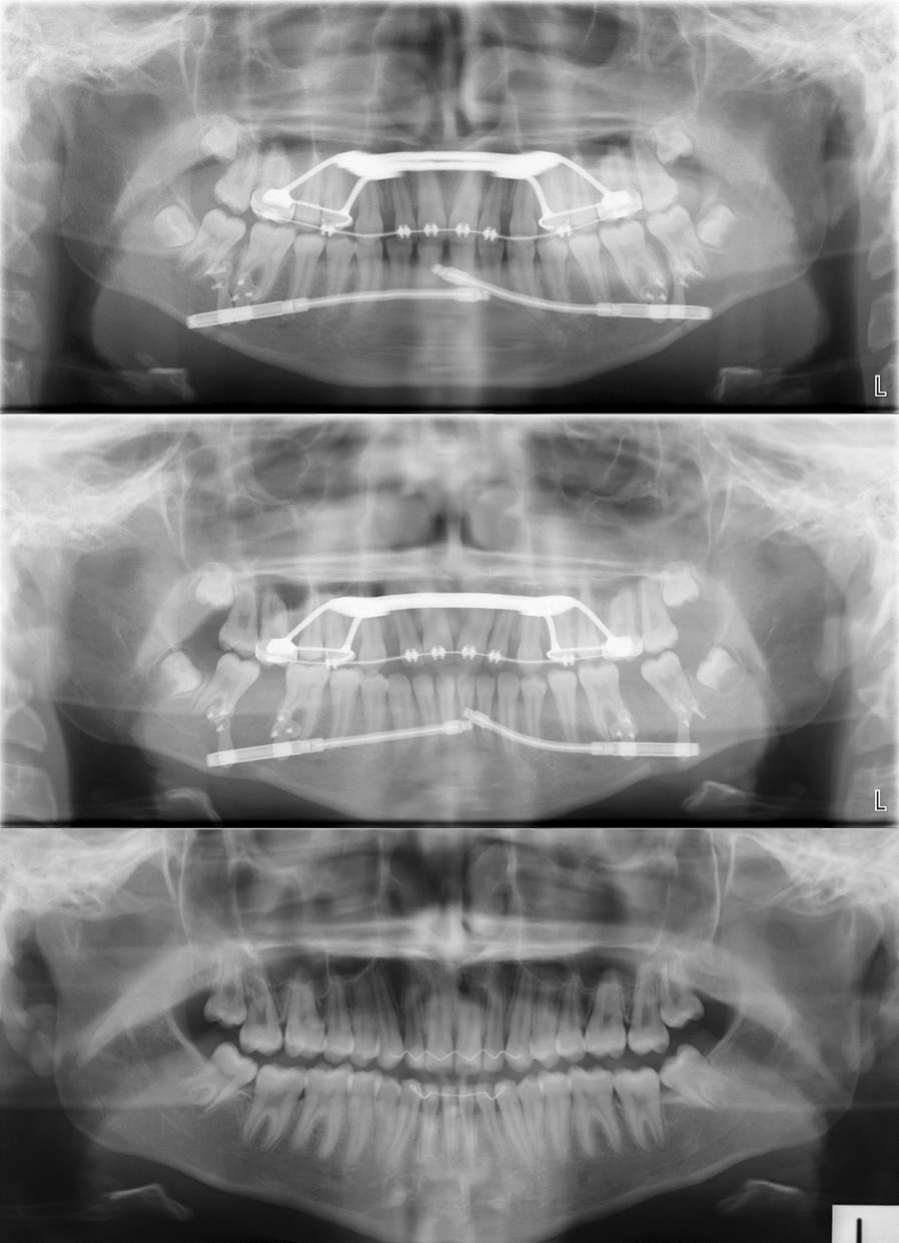
Orthopantomograms of subject #08 at T0, T1 and T2 showing the distractors, amount of distraction and consolidation of the mandibular body, respectively

Subsequent to the completion of the distraction and removal of the distractors, an approximately 9-month dentoalveolar settling and bone remodelling phase was observed. During this settling phase, molar distalisation and vertical control was performed with a mandibular lip bumper as required (Fig. 5). Finally, upon completion of the settling phase, routine orthodontic treatment and retention were performed.

### Radiographic analysis

**Fig. 6:**
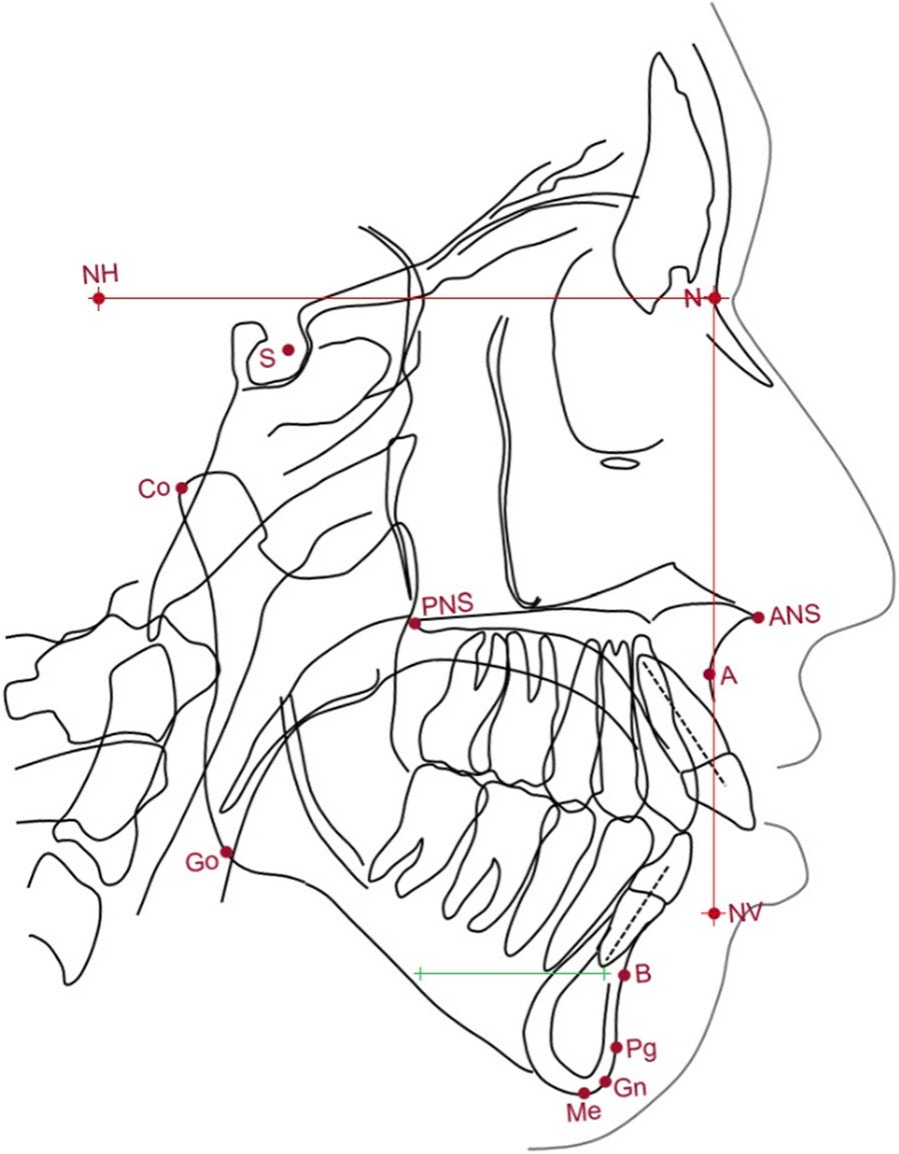
Diagram of the cephalometric landmarks and axes. The reference axis is represented in red and anterior mandibular axis in green

The low-dose spiral CT radiographs from T0 and T1 were imported into SimPlant O&O (V3.0.0.59) by Materialise Dental to produce a constructed lateral cephalogram. The constructed lateral cephalograms were orthogonally synthesized, which has been shown to be comparable to a traditional lateral cephalograms [29]. The constructed lateral cephalograms and T2 lateral cephalogram were standardised to 50 pixels per centimetre using Adobe Photoshop CS4. The calibrated constructed T0 cephalogram was standardised to a clinically orientated natural head position; a 10cm x 10cm reference axis was positioned upon T0 Nasion to represent the natural horizontal and vertical axes; and a 3cm anterior mandibular axis was placed within the mandible parallel to the natural horizontal plane (Fig. 6).

**Fig. 7:**
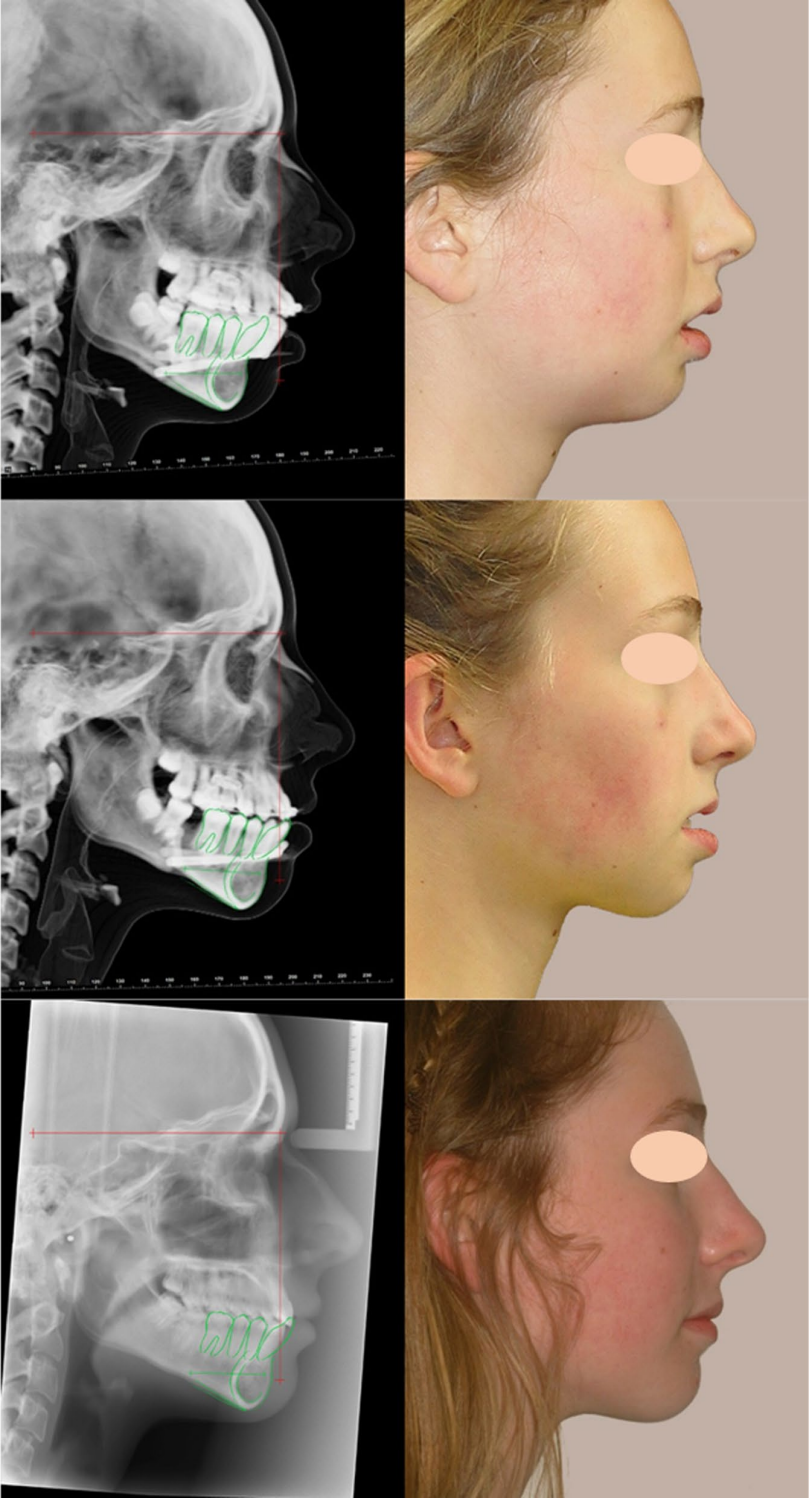
Lateral cephalograms with the reference axis (red) and anterior mandibular tracing with the anterior mandibular axis (green) and profile photographs of subject #08 at T0, T1 and T2

The subsequent constructed T1 and T2 radiographs were then superimposed upon the cranial base and the reference axis transferred to the T1 and T2 radiographs. For this study, T0 Nasion was defined as the (0,0) coordinate for all radiographs. The vertical axis was defined as the y-axis with positive values superior and negative values inferior. The horizontal axis was defined as the x-axis with positive values anterior and negative values posterior. The standardised radiographs were then imported into Quick Ceph and landmarks identified (Fig. 7). The maxillary plane (ANS-PNS), mandibular plane (GoMe) and lower facial height (ANS-Me) were constructed.

The T0 anterior mandible and anterior mandible axis was manually traced and cephalometric landmarks located to allow assessment of bending of the distraction callus. The T0 anterior mandible tracing was then superimposed upon the T1 and T2 anterior mandibles, and the axis rotation was measured relative to the horizontal plane (Fig. 7). The tracing and mandibular landmarks were superimposed on the T1 and T2 radiographs to facilitate consistent landmark localisation. A clockwise rotation of the anterior mandibular axis was defined as positive and counter-clockwise rotation negative.

Twenty-five per cent of the sample was remeasured a week later to assess landmark identification reproducibility and radiographic superimposition. The expert was found to have an intraclass correlation coefficient (ICC) measure for all landmarks over 0.9, indicating very good agreement.

### Statistical analysis

Descriptive statistics of the cephalometric landmarks and measurements between timepoints were calculated and presented as mean ± standard deviation. The Michigan growth study data served as a control group to compare post-surgical growth outcomes [28, 30]. It is recognised that the Michigan growth study based the assessment on chronological age, and there are studies questioning the efficacy of the CVM method so the growth comparison [31, 32].

## Results

**Fig. 8:**
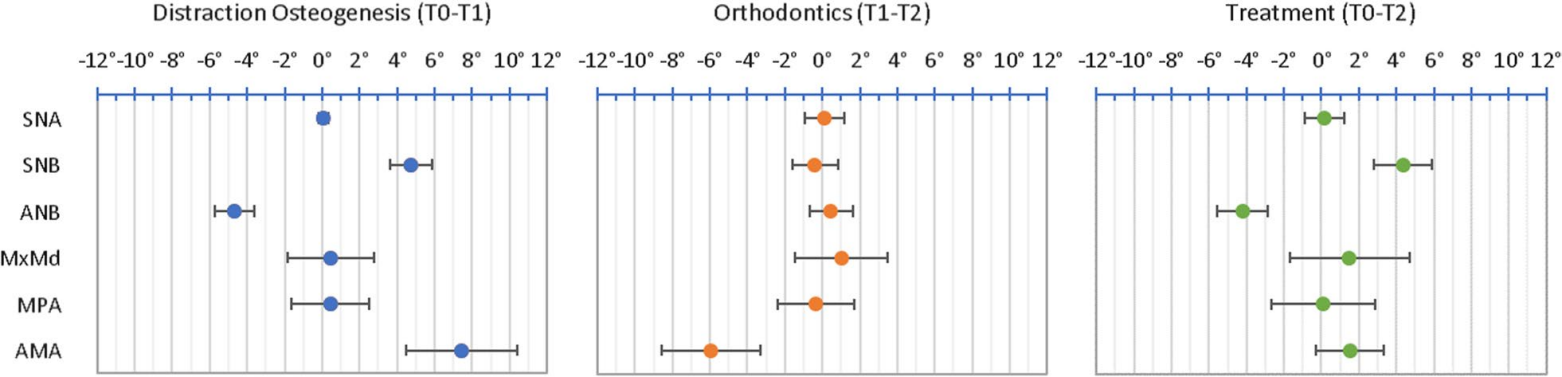
Mean changes in cephalometric angles with standard deviation error bars between T0–T1, T1–T2 and T0–T2

**Fig. 9:**
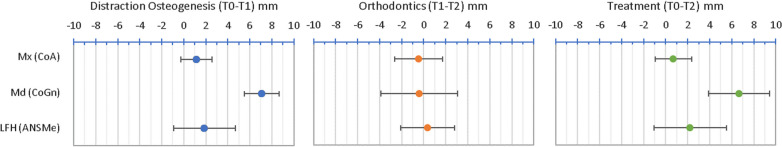
Mean changes in cephalometric linear measurements with standard deviation error bars between T0–T1, T1–T2 and T0–T2

**Fig. 10:**
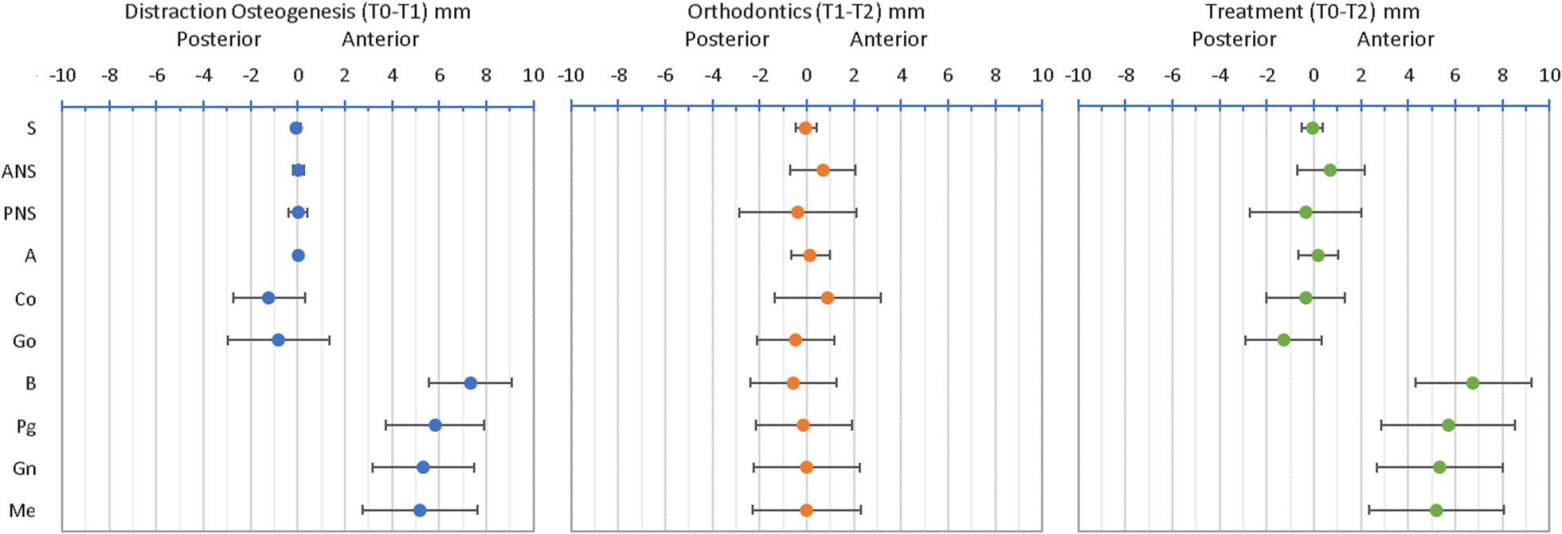
Mean horizontal changes (x-axis) in cephalometric landmarks with standard deviation error bars between T0–T1, T1–T2 and T0–T2

**Fig. 11:**
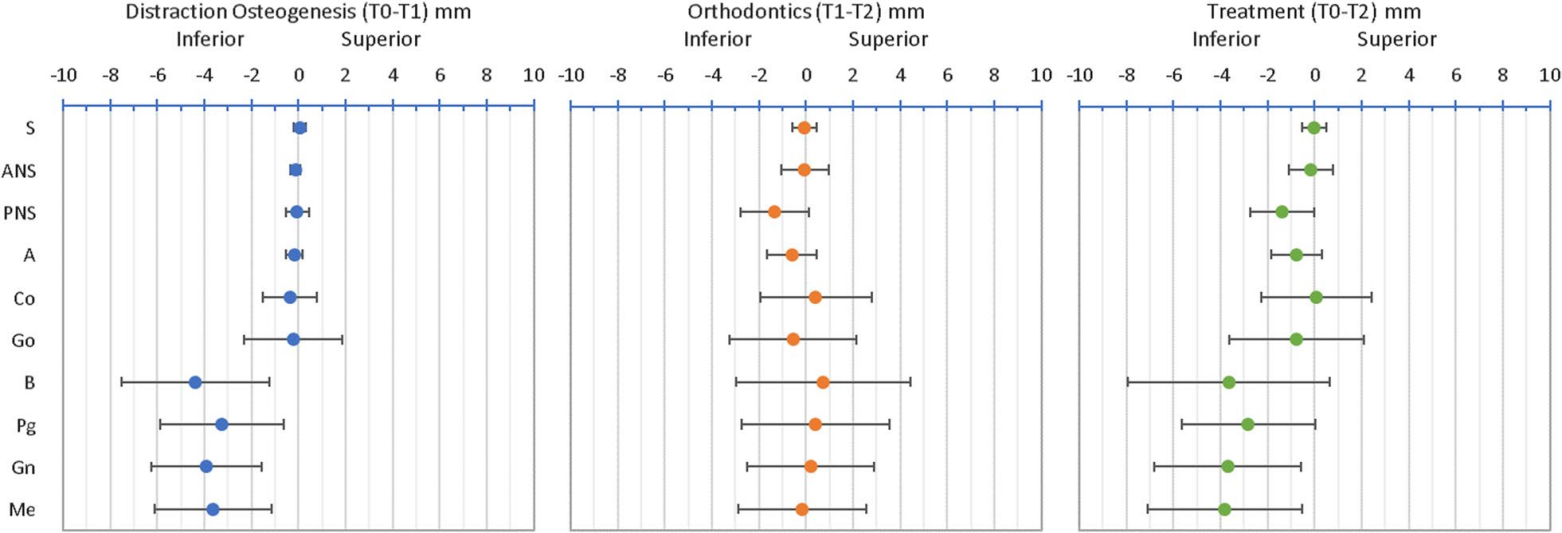
Mean vertical changes (y-axis) in cephalometric landmarks with standard deviation error bars between T0–T1, T1–T2 and T0–T2

Descriptive statistics of the craniofacial measurements between each time interval (T0–T1 and T1–T2) and the entire treatment (T0–T2) were calculated. The angular changes are presented in Table 2 and Figs. 8, 9, 10 and 11. The SNB angle increased with distraction and remained highly stable post-orthodontics. The ANB angle decreased to within the normal range and remained in this range following orthodontics. The mandibular plane angle (MPA) increased initially and decreased after orthodontics returning to its initial value. The anterior mandibular axis (AMA) rotation showed a clockwise rotation of 7.47 ± 2.96° with distraction and returned to near its original orientation during the orthodontic period.

The linear measurements are represented in Fig. 9. Mx (CoA) can be seen to increase in length with distraction. This was due to the posterior displacement of Co. Md (CoGn) significantly increased with distraction and on average remained stable following orthodontics. Lower facial height increased with distraction and was retained throughout treatment.

Figures 10 and 11 display the horizontal and vertical changes in the cephalometric landmarks. The posterior mandibular points (Co and Go) were posteriorly displaced with the distraction and then Go remained at this new position, while Co returned towards its initial position. The posterior mandibular points were fairly stable vertically during distraction and Go moved inferiorly following orthodontics. The anterior mandibular points (B, Pg, Gn and Me) moved anteriorly with distraction and then remained at this new position. The anterior mandibular points moved inferiorly with distraction and then were likely to remain at this new position.

The posterior displacement of the condyle (Co) during distraction (T0–T1) was not associated with changes in mandibular length (CoGn) at follow-up (T1–T2) showing a coefficient of 0.95, standard error of 0.54 and *p* value of 0.09.

The anterior mandibular axis rotated clockwise with distraction (T0–T1) and was associated with an inferior displacement of Me and increase in lower facial height (Table 2). Within the orthodontic phase (T1–T2), the counter-clockwise recovery of the anterior mandibular axis towards its original inclination was not however associated with movements in the anterior mandibular landmarks.

The increase in lower facial height between T0–T1 and T1–T2 was shown to be associated with the inferior displacement of the anterior mandibular landmarks over the same time interval.

There was no association between age at T1 and change in mandibular length (CoGn) between T1 and T2 (*p* value 0.585). There was no association between age at T1 and change in lower facial height between T1 and T2 (*p* value 0.518). When adjusted for the expected growth potential based upon the Michigan untreated Class II control [28, 30] between the post-distraction time interval (T1–T2), no association was found for lower facial height (*p* value 0.872) or mandibular length (CoGn) (*p* value 0.251). Assessment of growth following distraction compared to the Michigan Class II controls showed a reduction in mandibular growth following distraction. There was no association between the post-distraction time interval (T1–T2) and any of the cephalometric measurements (Table 2).

### IMDO complications

Like all surgical procedures post-operative pain and swelling occurs following IMDO. Pain and swelling remains during the distraction phase and alleviate within the 2 weeks following removal of the distractions. The most common complication of IMDO is infection of one of the distractor arms. Infection occurred in approximately half of the patients and was treated with a single course of oral Amoxicillin. Temporary paraesthesia occurred in one case immediately post-operatively and returned to normal within the first 2 weeks of distraction. The anterior distractor arm also became embedded within the lip of one patient and required repositioning out of the lip and was tied with floss to the central incisor to hold it away from the lip.

## Discussion

Growing patients may present with dentofacial deformities necessitating surgical correction [31]. Surgical intervention in growing patients has been largely discouraged because of the belief that further growth might contribute to correction of the malocclusion; surgical intervention may worsen the growth pattern; or inhibit future growth [33]. In 97% of cases, no significant change in skeletal relationship will occur after 5 years of age and thus the discrepancy which exists in early life will persist into adulthood [34–41]. For this reason, it is highly unlikely that a severe skeletal discrepancy will be resolved with residual growth [34–41]. Certain functional, aesthetic and psychosocial factors may necessitate early surgical intervention [42]. Both the surgical and growth factors may affect the outcome of the patients treated during growth [43].

The stability of BSSO mandibular advancement surgery has been shown to be highly stable [19]. Any new technique for surgical advancement of the mandible must be compared to the current gold standard technique. Within this study, IMDO appeared highly stable within a follow-up period of 2.3 ± 0.9 years with the ANB angle maintained within the normal range and the mandibular length remaining at the increased length. The large variation of B point in the vertical dimension may represent errors in landmark identification [41, 43–48]. Landmarks have been shown to demonstrate different fields of error in the horizontal and vertical especially when based upon curved surfaces [44, 46, 47, 49–54].

The condyle was displaced posteriorly during IMDO and returned towards its initial position in follow-up. During BSSO mandibular advancement the condyle is often also displaced posteriorly and then recovers anteriorly [55]. As in this study, the recovery of the condylar position following BSSO was also not associated with surgical relapse [55].

The anterior mandibular segment underwent clockwise rotation during distraction and recovered to near its pre-distraction angulation during consolidation and remodelling. This clockwise rotation was related to an increase in the mandibular plane angle and likely represents the effect of muscular forces and gravity upon the anterior segment resulting in bending of the distraction regenerate. Some judicious use of elastics to control the vertical were employed if required. An increase in the lower facial height of 1.88 ± 2.81mm also occurred during distraction and was maintained during the follow-up period. The anterior segment rotation underwent recovery towards its initial angulation; this was most likely caused by remodelling of the anterior mandible as it returned towards the patient’s initial mandibular plane angle.

Proffit et al. [12] have reported greater post-surgical relapse after surgical advancement of the mandible with BSSO in individuals with significant residual growth and recommended a cautious approach in such circumstances. IMDO was shown to respond like BSSO mandibular advancement in that subsequent mandibular growth was inhibited [56, 57]. Following IMDO growth appears to be inhibited in both the anteroposterior and vertical planes. Even though growth was inhibited it must be understood that the growth potential of untreated Class II patients is decreased relative to Class I patients [28]. Previous studies of distraction osteogenesis in growing patients have shown growth potential remains following the surgical correction but may not return to the untreated potential [58–60]. A limitation of this study relates to the uses of a historical control group in growing individuals. The Michigan growth study represents data that may be susceptible to secular trends [28, 30]. Given the limited amount of growth and high stability of the surgical correction, IMDO does provide advantages in the treatment of growing severe Class II patients.

Careful consideration of the advantages and disadvantages of IMDO versus a BSSO should be considered when clinicians are deciding which technique to undertake. The advantages of IMDO include the ability to control the exact amount of skeletal and asymmetrical correction through a controlled titrated advancement; a distraction site within the lower dental arch for utilisation in traditional mechanotherapy for decompensation; and by performing during growth removes the dilemma of treating immediately with camouflage or waiting for the cessation of growth for surgical correction. The disadvantages of IMDO include the potential for damage of the first or second mandibular molar, although this did not occur within our sample; the requirement of protraction of the mandibular second and possibly third molar through the newly formed bone; and possible clockwise distal segment rotation during distraction causing an increase the anterior mandibular clockwise rotation.

Within this study, IMDO was shown to be an affective technique for the treatment of mandibular deficiency. No neural disturbances were reported within this sample; however, further research into the neurological advantages of IMDO must be performed before any conclusions may be drawn. This is consistent with previously published data [61, 62]. The skeletal stability of the procedure resulted in an ideal skeletal relationship being maintained in all patients based upon ANB. Changes in condylar position during distraction showed no long-term effect upon stability of the surgical correction. A slight increase in lower facial height must be expected when completing this technique. IMDO was shown to respond like BSSO mandibular advancement in that subsequent mandibular growth was inhibited [12, 55, 56]. While the severity of the surgical correction required did not affect the stability, clinicians must still be cautious when performing surgical correction in patients with significant residual growth, as post-surgical changes may result in final chin position outcomes that are more retrusive than desired. Moreover, clinicians have to consider additional factors related to decision making when earlier distraction is chosen versus an acute mandibular surgical procedure post-adolescence. Patients may gain some additional psychological benefit from early intervention, but this must be weighed up with the capacity for the patient to cope with the reported increase in discomfort associated with the distraction procedure and subsequent removal of the distractors [5, 30, 61].

## Conclusion

Within the limitations of this retrospective pilot study, the following conclusions were drawn:

- IMDO appears highly stable within a follow-up period of 2.3 ± 0.9 years with the ANB angle maintained within the normal range and the mandibular length remaining at the increased length.
- The condyle was displaced posteriorly during IMDO and returned towards its initial position in follow-up.
- The anterior mandibular segment undergoes clockwise rotation during distraction and recovers to near its pre-distraction angulation during consolidation and remodelling.
- The mandibular plane angle increased with IMDO and returns towards the initial value in follow-up.
- The increase in lower facial height seen following IMDO remained during follow-up.
- Growth appears to be inhibited by IMDO in patients with significant residual growth.

## Data Availability

The data underlying this article will be shared on reasonable request to the corresponding author.
